# No increase in GFAP and S-100B in very preterm infants with mild periventricular leukomalacia or intraventricular hemorrhage: a pilot study

**DOI:** 10.3325/cmj.2022.63.564

**Published:** 2022-12

**Authors:** Maša Koce, Aleš Jerin, Domen Plut, Vanja Erčulj, Lilijana Kornhauser Cerar, Stefan Grosek

**Affiliations:** 1 University Medical Center Ljubljana, Ljubljana, Slovenia; 2 Institute of Clinical Chemistry and Biochemistry, University Medical Center Ljubljana, Ljubljana, Slovenia; 3 Faculty of Pharmacy, University of Ljubljana, Ljubljana, Slovenia; 4 Institute of Radiology, University Medical Center Ljubljana, Ljubljana, Slovenia; 5 Rho Sigma Research & Statistics, Ljubljana, Slovenia; 6 Neonatology Section, Department of Perinatology, Division of Gynecology and Obstetrics, University Medical Center, Ljubljana, Slovenia; 7 Faculty of Medicine, University of Ljubljana, Ljubljana, Slovenia

## Abstract

**Aim:**

To determine the serum levels of glial fibrillary acidic protein (GFAP) and S-100B in very preterm infants with and without periventricular leukomalacia (PVL) and/or intraventricular hemorrhage (IVH).

**Methods:**

The study enrolled preterm infants born between 23 and 32 weeks of gestation admitted to the Neonatal Intensive Care Unit, University Medical Center Ljubljana. PVL and IVH were determined with cranial ultrasound. Peripheral blood was collected in the first 24 hours after delivery and once between days 4 to 7. GFAP and S-100B concentrations were measured in serum samples. Infants with PVL or IVH were compared with infants without PVL or IVH.

**Results:**

Of 40 patients (mean gestational age 29.4 weeks), 7 had IVH and/or PVL. S-100B was detectable in peripheral blood in all patients at every measurement. In the group with IVH or PVL, the median S-100B at the first sampling was 0.43 (IQR 0.29-0.60) ng/mL, and 0.40 (IQR 0.33-1.01) ng/mL at the second sampling. In the group without PVL or IVH, it was 0.40 (IQR 0.29-0.6) ng/mL at the first sampling and 0.43 (IQR 0.34-0.62) ng/mL at the second sampling. The median GFAP was 0 regardless of the group and sampling time. The groups did not significantly differ in serum GFAP or S-100B levels.

**Conclusion:**

Peripheral blood levels of GFAP and S-100B were not significantly increased in very preterm infants that developed PVL or IVH. The predictive value of GFAP and S-100B as biomarkers of neonatal brain injury should be further explored in a larger cohort of neonates with more extensive IVH or PVL.

With the increasing survival of very preterm infants in the last decades, the number of long-term neurological impairments is rising. This presents a challenge in daily practice since mild brain damage is difficult to identify. Brain imaging to rule out brain injury in premature infants is routinely performed with cranial ultrasound (CUS) (1,2). However, mild brain abnormalities sometimes remain undetected until discharge. In this regard, blood biomarkers could greatly aid in the identification of the infants at risk to develop perinatal brain injury.

Several neurobiomarkers have been proposed as predictors of perinatal hypoxic-ischemic brain injury. In particular, glial fibrillary acidic protein (GFAP) and protein S-100B have been extensively studied in the pediatric population (1-13).

GFAP is present exclusively in the nervous system, being primarily produced by astrocytes. In case of neuronal death, GFAP leaks from damaged cells into the surrounding biological fluids. Consequently, GFAP levels have been studied as a potential early biomarker of perinatal brain damage in term and preterm infants. In cord blood, increased GFAP levels were observed in term newborns with hypoxic-ischemic encephalopathy (HIE) as defined by magnetic resonance imaging (MRI) (10).

Another well-studied potential biomarker of brain injury is protein S-100B. S-100B is a homodimeric calcium-binding protein found mainly in glial cells in the central nervous system (CNS) but also in nonneuronal tissues, particularly adipocytes. Apart from calcium homeostasis regulation, it plays a major role in cell differentiation and proliferation. In case of brain injury, S-100B not only leaks out from damaged glial cells but is also actively secreted as a part of the proinflammatory-axis (14). In low concentrations, it exhibits trophic properties, however higher levels of S-100B have neurotoxic effects (15). After hypoxic-ischemic brain injury, S-100B was detected in different body fluids, among others in urine and saliva, which are easily collected and where it can be easily measured. S-100B levels can be longitudinally assessed, the measurements have good reproducibility, and the reference ranges for the pediatric population are known, all of which make S-100B a possible ideal neurobiomarker.

An increase in a single biomarker may indicate neuronal and glial injury, however, a simultaneous measurement of different neuron-specific proteins has shown a far more superior sensitivity and specificity (16,17). Previous studies showed that GFAP and S-100B were elevated in the serum of neonates with moderate to severe HIE, but the levels of these neurobiomarkers in mild IVH or PVL have not been studied. In this study, we examined serum GFAP and S-100B levels in very preterm infants with and without IVH or PVL. We hypothesized that any-stage PVL or IVH was associated with higher concentrations of GFAP and S-100B.

## Patients and methods

### Study design

This study prospectively enrolled preterm neonates admitted to the Neonatal Intensive Care Unit at the Department of Perinatology (Maternity Hospital), Division of Obstetrics and Gynecology, University Medical Centre Ljubljana (UMC Ljubljana). We enrolled preterm infants with a gestational age of 23 to 32 weeks born between October 2020 and June 2021. Infants with major congenital, genetic, or chromosomal abnormalities were excluded.

Informed written consent was obtained before enrolment from the parents or legal guardians of all infants. The study was approved by the National Medical Ethics Committee.

Neonatal brain injury was determined by CUS in coronal and sagittal planes, through the anterior fontanelle. The images were scored by neonatologists using the classification system by de Vries and Papile (18,19). CUS findings were additionally independently reviewed by a pediatric radiologist. All enrolled patients were divided into two categories for each outcome: preterm neonates without evident brain injury on ultrasound, and preterm neonates who developed either PVL or IVH, or both.

Maternal and neonatal records were reviewed, and demographic, clinical, laboratory, and imaging data were collected for each infant.

### Biomarker sampling and analysis

Venous blood samples were collected without an additive. Serum was separated after centrifugation (1.500 × g for 10 min), and aliquots were stored at -20 °C until analyses. All serum samples were analyzed in one batch at the Institute of Clinical Chemistry and Biochemistry, UMC Ljubljana. S-100B concentration was measured with an electrochemiluminescence assay (Cobas e411 analyzer, Roche Diagnostics, Mannheim, Germany) with a detection limit of 0.005 ng/mL. GFAP was assessed with sandwich ELISA immunoassays (BioVendor, Brno, Czech Republic), with a detection limit of 0.05 ng/mL.

### Statistical analysis

The normality of the distribution of continuous variables was assessed with the Shapiro-Wilk test. The continuous variables are presented as median and interquartile range (IQR) or mean and standard deviation (SD). Categorical variables are presented as absolute counts and percentages. A 95% confidence interval (CI) was calculated for the area under the curve (AUC) when testing the threshold value of S-100B that divides the two patient groups. The correlation between successive S-100B and GFAP measurements was assessed with the Spearman correlation coefficient. Between-group differences in median peak GFAP and S-100B levels were determined with the Mann-Whitney U test. The two groups of infants and mothers were compared with the likelihood ratio test for categorical and with the Mann-Whitney U test or *t* test for continuous variables, as appropriate. A *P* value <0.05 was considered statistically significant. Statistical analysis was performed with SPSS, version 28.0. (IBM Corp. Armonk, NY, USA).

## Results

### Patients’ characteristics

The study enrolled 40 preterm infants (22 female) (Table 1). The groups of mothers were comparable in all characteristics, except in the use of prenatal antibiotics (*P* = 0.034). All mothers (n = 7) of infants with IVH/PVL and 23 (69.7%) mothers of infants without IVH/PVL received prenatal antibiotics (Table 2).

**Table 1:** Characteristics of infants with and without periventricular leukomalacia (PVL) and intraventricular hemorrhage (IVH)*

	Infants			
	total (n = 40)	without IVH/PVL (n = 33)	with IVH/PVL (n = 7)	P value
Male sex, n (%)	18 (45)	16 (48.5)	2 (28.6)	0.328^†^
Birth weight, mean (SD) in g	1249.8 (330.4)	1219.7 (345.7)	1391.4 (208.7)	0.216^‡^
Gestational age in days, median (IQR)	205 (197-216)	205 (196-216)	210 (206-216)	0.309^§^
APGAR at 1 min, median (IQR)	7 (6-8)	7 (6-8)	8 (7-9)	0.192^§^
APGAR at 5 min, median (IQR)	8 (7-9)	8 (7-9)	9 (7-9)	0.401^§^
Hospital stay length in days, median (IQR)	42 (35-54.5)	42 (35-55)	43 (33-50)	0.702^§^

*Abbreviations: SD - standard deviation; IQR – interquartile range.

†sequential probability ratio test.

‡t-test for independent samples.

§Mann-Whitney U test.

**Table 2:** The characteristics of mothers of infants with and without periventricular leukomalacia (PVL) and intraventricular hemorrhage (IVH) (likelihood ratio test for categorical values)*

	Infants			
	total (n = 40)	without IVH/PVL (N = 33)	with IVH/PVL (N = 7)	P value
Age, median (IQR) in years	29 (25-34)	29 (25-34)	28 (27-36)	0.630^†^
Placental abruption, n (%)	9 (22.5)	7 (21.2)	2 (28.6)	0.679
PPROM, n (%)	9 (22.5)	7 (21.2)	2 (28.6)	0.679
Chorioamnionitis, n (%)	6 (15)	5 (15.2)	1 (14.3)	0.953
Prenatal MgSO_4,_ n (%)	35 (87.5)	29 (87.9)	6 (85.7)	0.877
Prenatal antibiotic, n (%)	30 (75)	23 (69.7)	7 (100)	0.034
Prenatal betamethasone, n (%)	32 (80)	26 (78.8)	6 (85.7)	0.667
Vaginal birth, n (%)	16 (40)	12 (36.4)	4 (57.1)	0.313
Singleton pregnancy, n (%)	23 (57.5)	19 (57.6)	4 (57.1)	0.983

*Abbreviations: SD – standard deviation, PPROM – preterm premature rupture of membranes.

†t-test for independent samples.

### GFAP and S-100B in very premature infants

The groups did not significantly differ in GFAP or S-100B at any of the measurement points (Table 3). GFA*P* values were below the detectable concentration in 24 (60%) infants at the first sampling and in 25 (62.5%) infants at the second sampling. The area under the receiver operating characteristic (ROC) curve for on S-100B did not significantly differ from 0.50, which indicates that based on S-100B value it is not possible to differentiate between groups, both on the first and at the second sampling (Table 4). Therefore, S-100B had no predictive power outside the observed data and it cannot be used to distinguish between patients according to IVH or PVL.

**Table 3:** Glial fibrillary acidic protein (GFAP) and S-100B concentrations in infants with and without periventricular leukomalacia (PVL) and intraventricular hemorrhage (IVH) (Mann-Whitney U test)

	Infants without IVH/PVL (n = 33)	Infants with IVH/PVL (n = 7)	P value
Concentration, median (IQR) in ng/mL			
GFAP, sample 1	0 (0-0.05)	0 (0-0.05)	0.485
GFAP, sample 2	0 (0-0.05)	0 (0-0.05)	0.063
S-100B, sample 1	0.40 (0.29-0.60)	0.43 (0.33-1.01)	0.382
S-100B, sample 2	0.43 (0.34-0.62)	0.40 (0.35-0.49)	0.553

*Abbreviations: IQR - interquartile range

**Table 4:** The area under receiver operating characteristic (ROC) curve (AUC) for S-100B

S-100B	AUC (95% confidence interval), ng/mL
Sample 1	0.61 (0.4; 0.82)
Sample 2	0.42 (0.23; 0.62)

*Abbreviations: AUC – area under the curve

## Discussion

The present study, based on 40 very preterm infants, demonstrated that mild PVL or IVH in preterm infants were not significantly associated with serum GFAP or S-100B levels in the first 7 days after birth. These findings suggest that GFAP and S-100B may not represent reliable early biomarkers of mild PVL or IVH in very to extremely preterm infants. The lack of increase in serum levels of GFAP and S-100B in mild IVH or PVL may be due to our patients having scarce brain injury and inflammation. As described earlier, GFAP and S-100B are released from damaged glial cells after hypoxic-ischemic events in the perinatal period. If the damage is not extensive, the ensuing inflammation is not substantial, and the blood-brain barrier may remain intact.

Studies that investigated GFAP and S-100B as biomarkers of perinatal brain damage in peripheral blood have shown promising results. Cord blood levels of GFAP were significantly increased in low-birth-weight infants with periventricular white matter injury (6). GFAP concentrations also correlated with the severity of the disease (6). Similar results were reported in term infants with HIE (16). In infants treated with hypothermia, GFAP predicted brain injury as determined by brain imaging (20).

S-100B was significantly higher in term infants with moderate or severe HIE when compared with healthy infants or those with only mild HIE (21). Moreover, S-100B at a cut-off of 8.5 ng/mL reached the specificity and sensitivity of 90% and 71%, respectively, as a predictor of brain injury in term newborns (21). Plasma S-100B levels positively correlated with the severity of HIE as observed on MRI, but they did not significantly correlate with the neurodevelopmental outcome. At a cut-off of 1.6 ng/mL, S-100B reached a 91% specificity and a 40% sensitivity as a marker of HIE in term infants (8). Increased S-100B was also detected in saliva and urine, in which S-100B at a cut-off of 0.7 ng/mL achieved a specificity of 94.6% and a sensitivity of 91.3% as a predictor of HIE in preterm infants (9,22,23).

The results of our study are partly consistent with the previous studies in which GFAP levels in cord blood were not significantly higher in infants with HIE stage II-III compared with healthy controls (7,24). A recent study showed no difference in serum GFAP between infants at high risk for HIE and controls (24).

A large three-cohort study on 221 preterm infants found a median cord blood GFAP level of 261.4 (range 104.1-2433.7) ng/L on day 1 after birth. The longitudinal levels of GFAP did not indicate any distinctive pattern in relation to GA, morbidities, or other clinical variables, including the presence of IVH (25). A lower reference median serum GFAP value of 0.91 ± 1.019 ng/L was recently determined in 64 healthy preterm infants (26). The same authors also determined the reference values for S-100B of 392.12 ± 389.5 ng/L (26).

There are several limitations to our study, the most obvious being a small sample size of 40 preterm infants with heterogenous gestational ages. Small sample size has been a major weakness of multiple biomarker studies in preterm infants. In addition, the average age of infants included in our study was 30 weeks, and only 8 infants were extremely preterm infants, which are at the highest risk to develop IVH or PVL. In our study group, only 3 out of 8 infants had moderate IVH or PVL, and none had severe IVH or PVL. This may explain why we did not observe increased GFAP and S-100B in the study group. Lastly, we measured GFAP and S-100B twice. The second sampling was only approximately determined, and we observed no temporal change in the serum levels of GFAP and S-100B. Up to this point, no sampling protocol for neurobiomarkers in newborns has been proposed. Neurobiomarkers determined in blood samples are subject to many artifactual and biochemical changes, which differ according to sampling methods, storing, and analyzing procedures. The question of whether these variables affect the result has not been addressed yet. As the time between sampling and biomarker measurement in this study was prolonged, and the stability of the studied neurobiomarkers has not been determined, the storage time may present an uncertain factor that potentially interfered with the results.

In conclusion, the results of our study add to the pool of knowledge on neurobiomarkers in preterm infants. Larger and multicenter trials are needed to validate the results and to identify the biomarkers that could help pediatricians identify infants at risk of IVH or PVL and make a timely therapeutic intervention.
